# Nylon 6,6 Waste Nanofiber Membrane for Produced Water Filtration: Experimental, Performance Modelling, Optimization and Techno-Economic Analysis

**DOI:** 10.3390/membranes13020224

**Published:** 2023-02-11

**Authors:** Nur Syakinah Abd Halim, Shafiq Mohd Hizam, Wan Mohamad Syameer Wan Suhaimi, Ahmad Syahmi Ahmad Farid, Puteri Nur Khaliesah Abd Rahman, Mohd Dzul Hakim Wirzal, Nonni Soraya Sambudi, Nik Abdul Hadi Md Nordin

**Affiliations:** 1 Department of Chemical Engineering, Universiti Teknologi PETRONAS (UTP), Seri Iskandar 32610, Malaysia; 2 Department of Chemical Engineering, Universitas Pertamina, Simprug, Jakarta Selatan 12220, Indonesia

**Keywords:** nanofiber membrane, waste materials, cross-flow microfiltration, modelling, techno-economic analysis

## Abstract

Produced water (PW) is a by-product of oil and gas extraction, of which it is deemed as the primary contributor of wastewater stream in oil production. Conventional treatment such as membrane separation is favoured due to its sustainability and cost effectiveness. On the other hand, oceanic litters such as abandoned fishing nets endangered the marine life ecosystem, despite of its potential to be raw material for fabrication of nanofiber membrane (NFM). This study explores the potential usage of electrospun nylon 6,6 waste NFM for treatment of real PW. In terms of modelling, it is found that feed concentration is the dominant factor with R2 of 0.94 for permeate concentration response and 0.91 for average flux response. Moreover, the optimized system with average flux of 216.5 L/m^2^h with low specific power consumption of ca. 0.09 kWh/m^3^ is proven to be economically feasible with less than 5% error from predicted model. As for technoeconomic analysis, it is found that permeate flux plays the major role in controlling total capital cost (CAPEX) and operating cost (OPEX) of the system. The lowest total CAPEX and OPEX to achieve 10 ppm of permeate concentration, also was found to be RM 3.7 M and RM/year 1660, hence proving the economic feasibility of the proposed system.

## 1. Introduction

Produced water (PW), a by-product from oil and gas production contributes to the largest wastewater generated from the industry [1,2]. It is reported that PW were produced around 3–7 bbl per barrel oil in several wells in the US [3,4] and the PW volume can reach ten times of the oil [5]. Moreover, foaming occurrence dampens the oil and gas industry as it affects the oil and water separation, subsequently creating more PW waste [6]. Normally, conventional treatments such as coagulation/flocculation [7,8,9], adsorption [10,11,12], hydrocyclone [13,14,15] and floatation [16] are used either as standalone or in combination for PW treatment. However, they have few drawbacks such as low efficiency, corrosion, high cost operation, generating secondary pollutants and unable to completely remove microns or submicron sized oil droplets [17,18].

Henceforth, membrane filtration technique in PW treatment has been favoured due to several advantages such as low energy usage, chemical and mechanical stability, small footprint and provides comprehensive and integrable process methods [19,20,21]. Moreover, it has reliability in handling small droplets emulsion [22,23] as the pore size can be tuned into sub-micron size to ensure complete removal of oil droplets [24,25]. Apart from that, in order to meet the discharge requirements, membrane is usually be applied as the additional step for conventional treatment [17]. One of the most common membrane filtrations for PW treatment is using thermal desalination technique such as membrane distillation (MD). MD operates based on the thermal difference between the feed and permeate which is separated by hydrophobic membrane layer. MD can be a crucial separation component for the PW treatment because of its high water recovery [26]. Nevertheless, the energy requirement particularly sensible heat is considerably high hence reducing its competitiveness with conventional PW treatment. Another method by Ozgun et al. (2013) integrates nanofiltration—reverse osmosis (NF-RO) using polyamide membrane for PW treatment [27]. The research reveals superb flux of up to 100 L/m^2^ h, although NF and/or RO are more susceptible for membrane fouling due to the usage of high pressure [28]. Study conducted by Mohd Hizam et al. (2020) suggested forward osmosis (FO) is suitable to treat PW with air sparging as a means of fouling control [2]. While it was claimed to have a flux enhancement up to 1.63× (from 9.49 to 15.48 L/m^2^ h), FO suffers from reverse salt flux phenomena which can cause a concern.

Nanofiber membrane (NFM) has received major limelight due to its high porosity, huge specific surface area to volume ratio, small pore size and its ability in handling fouling problem [19,29,30,31,32,33]. It is usually produced in a form of mat fibres and has fibres with diameters of few hundreds nanometres to a several microns [34,35]. There are several ways to fabricate NFM such as melt blowing [36,37], phase separation [38,39], self-assembly [40,41] and electrospinning [42,43]. Electrospinning is highly preferable due to its simplicity and able to fabricate membrane with very thin fibres [17,44]. Such a study conducted by Abd Halim et al. (2019) studied on the application of solvent vapor treatment on nylon 6,6 NFM membrane for PW filtration [45]. It was found that solvent vapor treatment was able to improve membrane tensile strength while also reducing fouling propensity when treating PW. Moreover, the NFM used amount to low specific power consumption of 0.45 kWh/m^3^, which makes it economically attractive.

However, one of the major disadvantages of using NFM is membrane fouling. Normally, membrane fouling is affected by the feed composition, major components concentration, water chemistry and membrane properties. Furthermore, due to its non-woven structure, NFM has a rougher surface, in which foulant are prone to entrapment. Since the membrane performance is affected by membrane fouling, the permeate flux will be reduced overtime. Hence, the amount of clean water produced per kilowatt hour (kWh/m^3^) of pump will be decreased. Therefore, it is important to analyse the data via analysis of variance (ANOVA) analysis to optimize the permeate concentration and flux.

By having excellent membrane properties and suitable feed parameters, it can control fouling rate (time, cross-flow rate and pressure), hence enable the production rate of clean water (energy usage/kg water) to be optimized. Thus, it is important to optimize the permeate concentration, flux and power consumption. Furthermore, for real case situation, some of the operating parameters such as feed concentration is hard to be controlled as it varies depending on the source collected. Hence, membrane properties such as membrane surface area plays an important role in controlling membrane performance. In this project, a process modelling is developed to optimize the aforementioned parameters. Through data simulation and validation, a solid prediction is obtainable, as well as developing model sensitivity via principal component analysis (PCA). In business perspective, the optimization data and PCA plays an important role for economic evaluation of the system. It is important to prove and compare the feasibility of the system with the existing membrane technology system.

Numerous studies on the technoeconomic analysis (TEA) of membrane filtration have been conducted for wastewater applications including for PW treatment. Osipi et al. (2018) studied on the TEA of desalination technologies for onshore (Brazilian onshore oilfield) PW treatment [46]. The aim of the study is to determine the most suitable routes and study on the new technologies’ limitations of retro-techno-economic analysis. In this study, few conventional and new technologies were combined such as forward osmosis (FO), reverse osmosis (RO), assisted RO, microfiltration (MF), mechanical vapor compression (MVC) and MD. It is found that MF-RO provides the cheapest desalination route for PW up to 90 g/L salinity. For cases of higher salt content, the most economical choice is by using MF-RO accompanied by assisted RO. In addition to that, Tavakolli et al. (2017) also reported on the economic feasibility of MD application for PW treatment focusing on Marcellus shale gas play located in West Virginia [47]. The model in this study was developed using combination of experimental data results, ASPEN Plus process model and cost estimation. It is reported that thermal energy cost gave major impact on total cost of treating PW in the MD plant.

In this study, a cross-flow microfiltration system using NFM made from fishing net lines (nylon 6,6) waste will be studied in terms of membrane characterization, system optimization and economic evaluation to understand the effects of feed concentration (%), flowrate (mL/min) and membrane area (m^2^) towards permeate concentration (ppm) and average flux (L/m^2^ h) of the proposed system. This study will highlight the novelty in usage of waste materials specifically nylon 6,6 waste obtained from disposable fishing net lines. Apart from that, it provides the economical insight of using waste materials for PW treatment.

## 2. Experiments Methodology

### 2.1. Preparation of Nylon 6,6 Waste Solution

The main materials were glacial acetic acid (99.85%, VWR Chemicals, Radnor, PA, USA), formic acid (98–100%, MERCK, Kenilworth, NJ, USA) and nylon 6,6 waste. The nylon 6,6 waste was obtained from fishing line waste. A mixture of formic acid and acetic acid with a ratio of 1:1 was used as solvents to dissolve nylon 6,6 waste (14.0 wt%). The solution was then stirred overnight until it became homogenous [48].

### 2.2. Electrospinning of Nylon 6,6 Waste NFM

A 5 mL syringe was filled with nylon 6,6 waste solution and it was attached with a capillary tip of a 0.7 mm inner diameter. The flowrate was set at 0.4 mL/h. The voltage used was 20.0 kV and the distance from needle tip to a metal screen collector was 15 cm. Aluminium foil was placed on the rotator and the speed of collector rotation was set at 500 RPM [45].

### 2.3. Membrane Characterization

Membrane characterization was based on surface morphology, functional group, hydrophilicity and porosity. Field Emission Scanning Electron Microscope (FESEM, Model: VPFESEM, Zeiss Supra55 VP, Feldbach, Switzerland) was used to observe the membrane surface morphology. The sample was coated with gold after being mounted onto a metal substrate. Fibre diameter and pore size were measured by using ImageJ Software (ImageJ 1.53e, Bethesda, MD, USA). Additionally, the functional group presence in the membrane was analysed by using Fourier Transform Infrared Spectroscopy (FTIR, Model: Thermo-Nicolet, Waltham, MA, USA) and compared with result from our previous reports [49]. Goniometer was used to measure contact angle via Sessile Drop Method (IFT, Model: OCA 20, Data Physics, Filderstadt, Germany). The contact angles were measured three times by using the build-in software Interfacial Tension (SCA 20, Filderstadt, Germany). Furthermore, porosity was measured by using dry-wet method in which the membrane weight and volume were measured and calculated [50].

## 3. Modelling and Technoeconomic Analysis Methodology

### 3.1. Prediction Model

**Figure 1:**
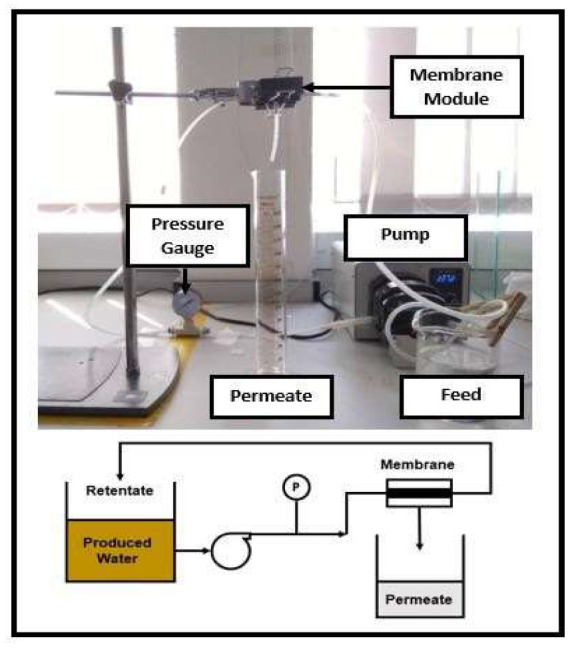
Cross-flow Microfiltration (MF) Testing unit.

In this study, JMP software (JMP Pro 14.0.0, SAS, Cary, NC, USA) was used to find a suitable model that best fits two responses which are permeate concentration (ppm) and average flux (L/m^2^ h) for cross-flow MF system as illustrated in Figure 1. Permeate concentration was determined using Ultraviolet-Visible (UV-VIS) Spectrophotometry (Model: Hach DR-6000, Hach Company, Loveland, CO, USA). After obtaining the suitable model equations, the prediction expression can be developed and later proved through sensitivity analysis via PCA and experiment validation. The experiments for cross-flow MF system consists of experiment parameters such as feed concentration (0–100%), feed flowrate (200–300 mL/min) and membrane area (9–18 cm^2^). Moreover, multiple regression was used to analyse the experimental data. To obtain the highest R^2^ values, the models were built based on quadratic and 2-factor variables, combined with backward elimination method. Later, the suitability of the final model was evaluated based on their adjusted R^2^ and their *p*-value.

**Figure 2:**
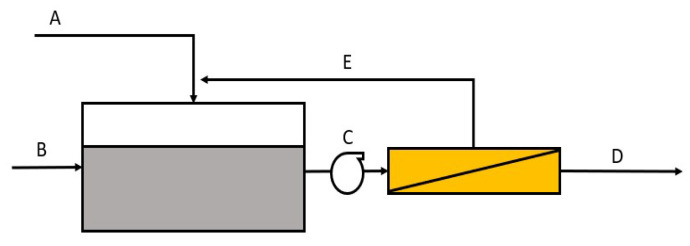
Scale-up model of cross-flow microfiltration (MF) system used in this study.

To further strengthen the validity on the chosen predicted expressions, mass balance was done using Microsoft Excel. Based on the generated predicted expressions, input of the concentration of feed in percent (%), feed flowrate (mL/min) and membrane area (m^2^) can be varied, and the issuance will be on the concentration of permeate and the resulting average flux in an hour. Prior to that, the mass balance will also provide information on the volumetric flowrate (m^3^/h) in each stream which is crucial for technoeconomic (TEA) analysis. The mass balance is based on Equations (1)–(6). Figure 2 shows the scale-up setup of MF system used in this study for techno-economic analysis.

Mass_in_ = Mass_out_ + Accumulation (1)

However, accumulation is assumed to be negligible. Therefore,

Mass_in_ = Mass_out_ (2)

Component balance is derived as follows:

X_in_ × Mass_in_ = X_out_ × Mass_out_ (3)

where:

X_in_ = mole fraction of X inlet streams;
X_out_ = mole fraction of X outlet streams.

A + B = D(4)

C = E + D(5)

A + B + E = D(6)

where:

A = Amount of water needed to dilute the produced water feed;
B = Amount of produced water feed;
C = Amount of produced water solution to be filtered;
D = Amount of permeate to be produced;
E = Amount of retentate to be recycled to feed stream.

### 3.2. Techno-Economic Analysis

After all the models have been analysed and constructed, a comparison is made between each model to select the best alternative to run a plant with the minimum (lowest) power consumption. The comparison of the capital expenditure, CAPEX (membrane module, storage tank for feed and permeate, pump) and operational expenditure, OPEX (energy consumption and membrane replacement) are then calculated and analysed using Microsoft Excel (Microsoft 365, Washington, DC, USA).

#### 3.2.1. List of Assumptions

Few assumptions and parameters must be made before performing the economic analysis. The list of the assumptions and parameters is shown in Table 1. Table 2 tabulated the factors included for CAPEX calculation as mentioned by Smith (2005) [51].

#### 3.2.2. Capital Expenditure (CAPEX)

Capital cost is a fixed cost (one-time expense) for asset purchasing such as pump, storage tank and membrane module. The equipment cost for pump; C_p_ can be calculated by using Equation (7) [52,53]. In addition to that, for the calculation of equipment cost, Equation (8) was used [51]. For the calculation of the physical plant cost (PPC), the equipment cost will be multiplied with the typical factors for capital cost estimation as shown in Equation (9) and Table 2. Lastly, the fixed CAPEX can be determined using Equation (10).

C_p_ = I × f_m_ × f_p_ × f_l_ × 81.27 × (Q × P)^0.4^ (7)

where:

I = cost index ratio for updating the cost to the recent year;
f_m_ = factor for pump construction material;
f_p_ = factor for suction pressure range;
L = factor for labour costs;
Q = pump flow capacity (m^3^/h);
P = pump outlet pressure (kPa).

The cost index, I can be obtained from United Nations Monthly Bulletin Statistics [52]. In this study, the cost index used is 125.2 based in Malaysia. As for f_m_, the material used is carbon steel, hence f_m_ = 1.0, meanwhile for f_p_, the pressure for microfiltration is below 10 bar, hence f_p_ = 1.0. The factor for labour cost is set at 1.4 since the cost required for labour to install equipment is 40% of the cost required [53,54].

C_E_ = C_B_ (Q/Q_B_)^M^ f_m_ f_p_ f_t._ (8)

where:

C_E_ = Equipment cost ($);
C_B_ = Base cost ($);
Q = Design capacity (m^3^);
Q_B_ = Base size;
M = Cost exponent;
f_m_ = Correction factor for material;
f_p_ = Pressure correction factor;
f_t_ = Temperature correction factor.

Physical Plant Cost (PPC) = C_p_ + C_E_ × (1 + f_1_ + f_2_ + f_3_ + f_4_) (9)

Fixed Capital Cost = PPC × (1 + f_5_ + f_6_ + f_7_) (10)

#### 3.2.3. Operating Expenditure (OPEX)

Operating cost is a cost required to run a specific operation and maintaining the business existence. Operating cost is divided into two types: fixed cost and variable cost. Fixed cost is the compulsory cost needed to run the operation such as raw material cost and utilities cost. Meanwhile, the variable cost is the cost which has inconsistent values depending on the demands and necessity of production such as maintenance. The operating cost in this study is based on annual utilities, U (Equation (13)) [52,54] and membrane replacement cost (Equations (14) and (15)). For membrane replacement cost, it is divided into two parts: membrane fabrication, M_F_ and membrane materials, M_M_. For the amortization rate of membrane, it will be based on 18 months of membrane life and 0.08 of interest rate [54]. The total operating cost (RM/y) is calculated based on the Equation (16).

Scale-up factor, SC

SC = Q_T_/Q_C_ (11)

where:

Q_T_ = Targeted clean water production rate (m^3^/h);
Q_C_ = Current permeate flow (m^3^/h).
Scale-up factor membrane area, SCM

SCM = Q_T_/Q_C_ × A_C_ (12)

where:

Q_T_ = Targeted clean water production rate (m^3^/h);
Q_C_ = Current permeate flow (m^3^/h);
A_C_ = Current membrane area (m^2^).

U = E_SP_ × Q_T_ × t (13)

where:

E_SP_ = Specific pump energy (kW/m^3^);
Q_T_ = Targeted clean water production (m^3^/h);
t = Plant operating hours (h/year).

M_F_ = (P × t_m_ × SCM × a)/A_m_ (14)

where:

P = Power supply high voltage (kW);
t_m_ = Time consumption to produce membrane (h);
A_m_ = Size of membrane produced (m^2^);
SCM = Membrane scale-up factor (m^2^);
a = amortization rate.

MM = ([(S_s_ × P_s_) + P_m_]/A_m_) × SCM × a (15)

where:

S_s_ = Amount of solvent used (g);
P_s_ = Price of solvent (RM/g);
P_m_ = Price of polymer used (RM).

Operating Cost = U + M_F_ + M_M_ (16)

## 4. Results and Discussion

### 4.1. Membrane Characterization

**Figure 3:**
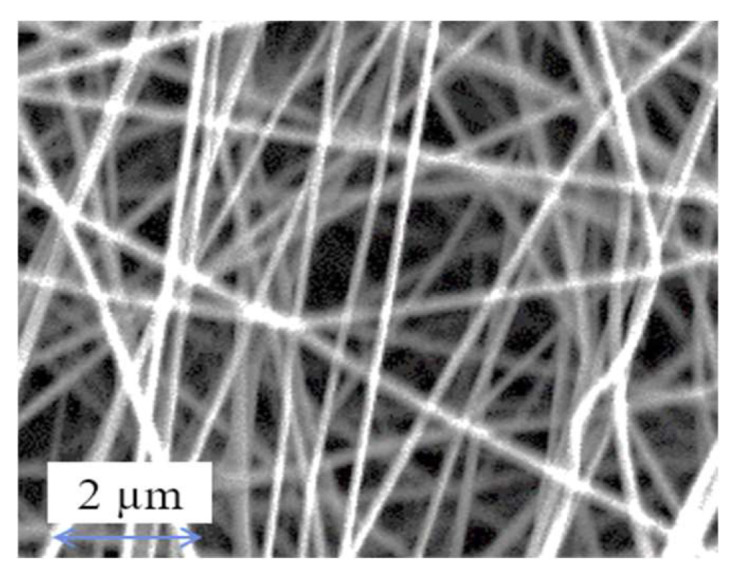
Surface morphology of nylon 6,6 waste NFM.

Figure 3 and Table 3 shows the surface morphology and membrane properties of nylon 6,6 waste NFM. Based on the FESEM image, the nanofibers were nonuniformly distributed and in cylindrical shape. Additionally, the size of the fibres formed were in nanometre range (50 nm to 1000 nm) which is around 104.65 ± 64.59 nm as shown in Table 3 [55]. Furthermore, the nylon 6,6 waste NFM has pore size approximately at 0.2 µm with high porosity of 81.34% which correlated with other literature (>80%) [45,56].

**Figure 4:**
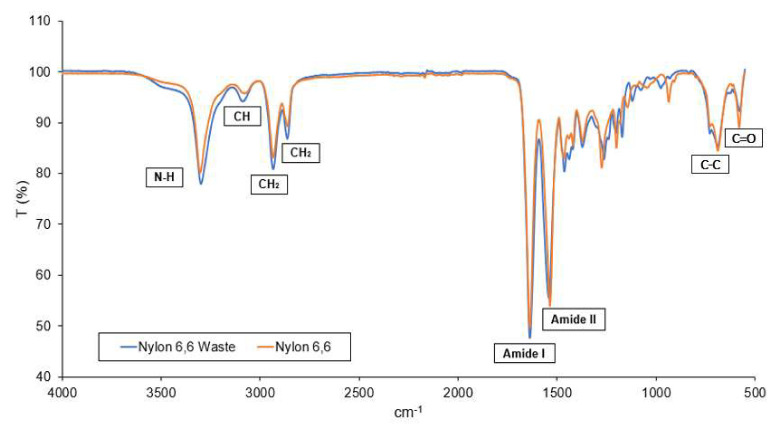
FTIR of nylon 6,6 waste NFM.

Figure 4 represents the FTIR spectra of waste and pure nylon 6,6 NFM. It is found that nylon 6,6 waste NFM has similar functional groups and characteristics peaks as to the pure nylon 6,6 NFM [49,57]. N–H stretching can be observed at peaks 3297 cm^−1^ (for nylon 6,6 waste) and 3301 cm^−1^ (for nylon 6,6). Meanwhile, CH_2_ stretch was represented by the peaks 2933 cm^−1^ and 2861 cm^−1^. Moreover, for both NFMs, amide–I band is represented at peak 1636 cm^−1^. In addition to that, the amide–II band is attributed at peaks 1540.64 cm^−1^ with, O=C–H and C–C bond attributed at 580 cm^−1^ and 685 cm^−1^ for both membranes, respectively. It can be concluded that the fishing net line used is a pure nylon 6,6 given that all the important functional groups of nylon 6,6 were present in the nylon 6,6 waste NFM.

**Figure 5:**
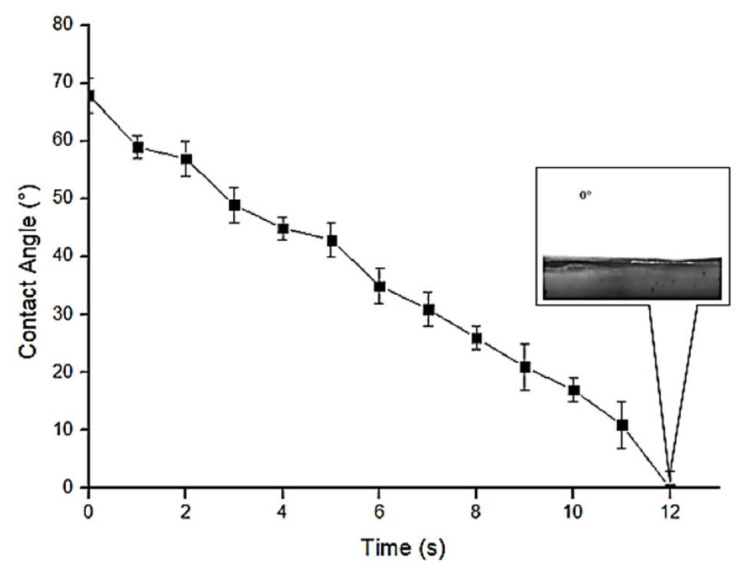
Contact angle of nylon 6,6 waste NFM.

Figure 5 shows the dynamic water contact angle (WCA) of nylon 6,6 waste based NFM. The WCA started at 69° and eventually reached 0° after 12 s. This is a common trend for hydrophilic membrane since intermolecular bonding is formed with water (i.e., hydrogen bond). Additionally, normally, hydrophilic membrane is more preferable to reduce fouling in membrane separation [58], given that membrane surface roughness usually lower when the membrane is hydrophilic (smooth surface) [45].

### 4.2. Prediction Model

#### 4.2.1. Permeate Concentration

**Figure 6:**
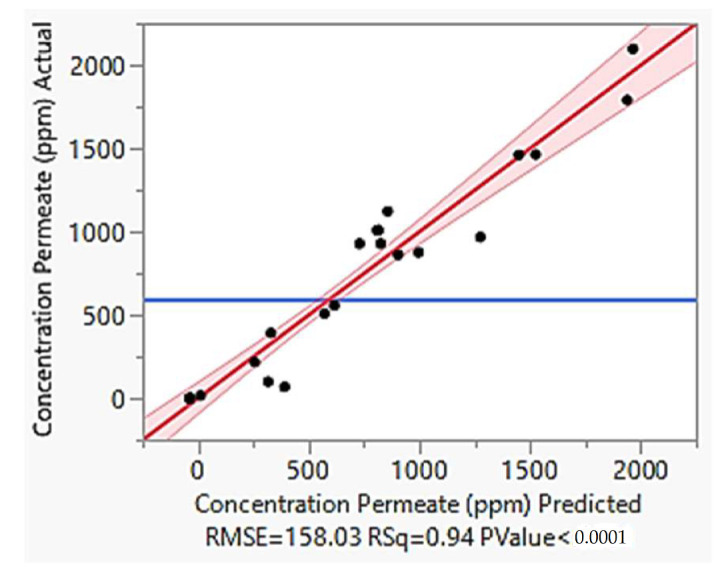
The linear-linear plot of actual and predicted permeate concentration.

By using backward elimination method, cubic cross variable equation model was the most suitable model for permeate concentration due to significant *p*-value of < 0.05 for all of the parameters (Table 4). Moreover, this model gives R^2^ value of 0.94 with root mean square error (RMSE) of 158.03 as shown in Figure 6 indicating the model significance.

Moreover, the significance of each parameter can also be observed on the logworth data value in Table 4. Based on the table, cross variable between feed concentration squared variable and area (C^2^A) has the highest log worth value of 8.540 followed by feed concentration squared variable (C^2^) with a logworth value of 7.603. The least significance parameter is C^2^F variable with a log worth value of 4.668. From this table, it can be concluded that concentration of feed plays the highest impact in determine permeate concentration.

Figure 6 shows the linear-linear plot between actual and predicted permeate concentration together with residual plot showing the random distribution of errors. Based on the figure, the model is highly reliable to predict the permeate concentration since it has high R^2^ value of 0.94 and RMSE of 158.03. Apart from that, the residual points are distributed evenly and close to the 45° normal line indicating the model is fitted well with the data [59]. By having normally and evenly distributed residual points, it implies that the data variation is sufficient for the model development [60]. Furthermore, the factors relative impact can be identified using the model equation by comparing the factor coefficients. Equation (17) shows the simplified predicted expression of the model.

Concentration Permeate

= −37.67321727 + 0.6852856225 C^2^ − 107.6731136 C^2^A − 0.002112486 C^2^F + 0.000224005 CF^2^ (17)

#### 4.2.2. Average Flux

**Figure 7:**
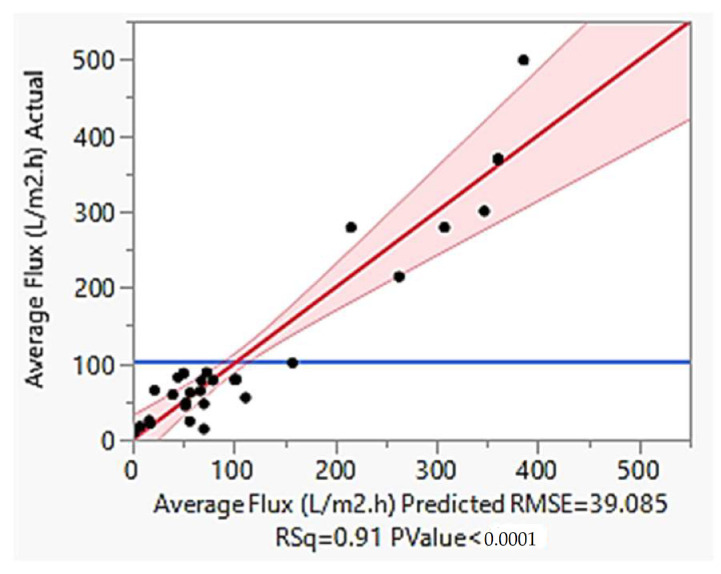
The linear-linear plot of actual and predicted average flux.

Cross variable cubic model was identified as the most suitable model for average flux since most of the parameters has significant *p*-value of < 0.05 as shown in Table 5. This model produces R^2^ value of 0.91 and the least RMSE of 39.085 (Figure 7) of which indicate the significance of the developed model.

Besides that, based on Table 5, it shows the significance of each parameter based on the log worth value, which later supported by the PCA (Section 4.4). Cross variable of feed concentration and area (CA) has the highest log worth value of 6.850 followed by cross variable between concentration of feed squared (C^2^: 4.979). Flow rate and area variable were the bottom two less significant variables with log worth value of 0.640 and 0.044 respectively.

Figure 7 shows the linear-linear plot between actual and predicted average flux together with residual plot showing the random distribution of errors. Based on the figure, the equation fits well with an R^2^ value of 0.91 and RMSE of 39.085. Moreover, most of the residual points are randomly distributed close to the straight line showing the reliability of the developed model. Equation (18) shows the simplified predicted expression of the model.

Average Flux

= 4528.7126134 + 23.948099025 C − 57.91071999 F − 17535.50124 A − 0.344946772 CF + 9540.1281791 CA − 1220.951209 FA + 0.227584301 C^2^ + 0.2655657131 F^2^ − 66.49475173 C^2^A + 0.0006537298 CF^2^ − 0.000701717 C^3^ − 0.000383848 F^3^ (18)

### 4.3. Membrane Performance

#### 4.3.1. Permeate Concentration

**Figure 8:**
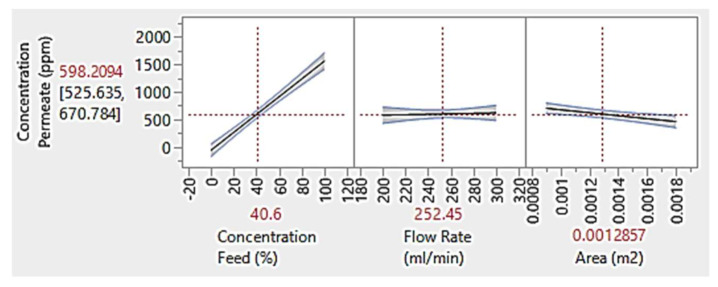
Graph of permeate concentration at constant feed concentration of 40.6%, flowrate of 252.45 mL/min and membrane area of 0.0013 m^2.^

Figure 8 illustrates the relationship between permeate concentration (ppm) with feed concentration (%), flowrate (mL/min) and membrane area (m^2^). Based on the figure, feed concentration is directly proportional to the permeate concentration. The higher number of solutes presents in the feed will give higher number of solutes presents in the permeate given that the feed flowrate and membrane area are kept constant. Meanwhile, greater membrane area gives more space (surface area) for the feed to fill in, hence gives greater number of functional membrane pores to filter the solutes from the feed. This will give lower number of solutes presents in the permeate [61]. However, as for the flowrate, it has subtle impact as compared to the others. Nonetheless, it was implied that higher flowrate gives lower permeate concentration. This can be explained by the cake layer formation when low flowrate is applied. Initially, as lower feed is applied, it allows more time for the solute to settle on the membrane surface, hence creating cake layer formation. In other words, higher feed flowrate would decrease the retention time of the foulant, hence controlling the fouling effect. The permeate concentration values were also tabulated in Table A1 in Appendix A.

#### 4.3.2. Average Flux

**Figure 9:**
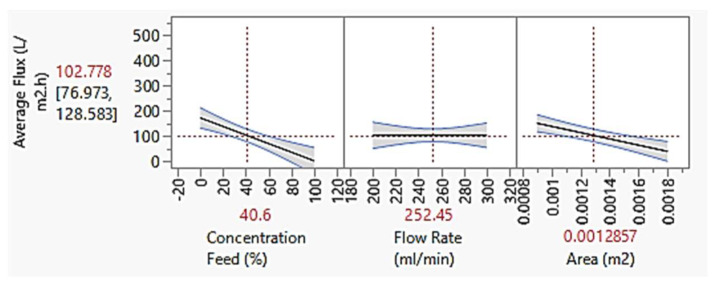
Graph of average flux at constant feed concentration of 40.6%, flowrate of 252.45 mL/min and membrane area of 0.0013 m^2^.

Figure 9 shows the relationship between the average flux with feed concentration, flowrate and membrane area. The average flux can be increased by reducing the feed concentration [62] and membrane area. This can be explained by lower fouling tendency when using lower feed concentration [63]. The lower amount of solute present in the feed reduced the number of membranes pores blockage, which hence allowing more permeate to pass through the membrane. Apart from that, lower membrane area also increases the average flux [64]. Given a constant feed concentration and flow rate, membrane with smaller area has faster flow rate and hence disallowed the solute from the feed to resettle on the membrane surface. Meanwhile, when using membrane with greater area, the solute has enough time to resettle on the membrane surface as the water movement is slower, hence causing cake layer formation. However, as for the flowrate, the effect was not as obvious as the others, same as in Figure 8. Nevertheless, it was assumed that higher flowrate gives lower average flux. When using membrane with lower flowrate, it allows transmembrane pressure to be created (more contact time between feed and membrane surface), hence more permeate will pass through membrane. The average flux values were also tabulated in Table A1 in Appendix A.

### 4.4. Principle Component Analysis

#### 4.4.1. Permeate Concentration

**Figure 10:**
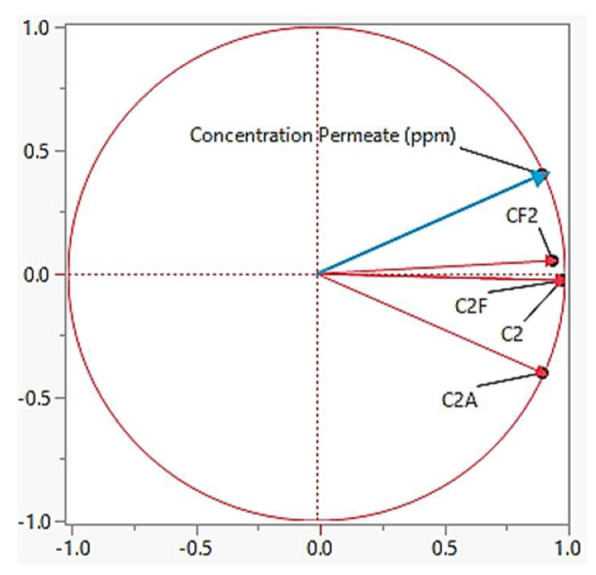
Principle component analysis for the permeate concentration.

PCA was performed to assess the correlation and dependency of permeate concentration towards feed concentration, flow rate and area. Figure 10 shows the PCA of the permeate concentration. Based on Figure 10, feed concentration (C) has positive correlation and has the highest impact towards the permeate concentration since the angle parameter between the objective function (concentration permeate—indicated by blue line) is below 90°. Theoretically, the component that is located below 90° is directly proportional to the responding variable. In other words, the higher the feed concentration, the higher the permeate concentration. This result is correlated with the data of the logworth value from Table 4 in which all variable consists of feed concentration (C). Nevertheless, when membrane area (A) was cross with feed concentration (C), the angle parameter is almost inclined towards more than 90°. In other words, the higher the membrane area, the lower the permeate concentration. This can be explained by the larger membrane space area which provides more available functional membrane pores which can filter a greater number of solutes presents in the water. In addition to that, Figure 10 also shows that flowrate is directly proportional towards the permeate concentration since it is located below 90^o^ when it was cross variable with C. The higher the flowrate, the lower the concentration permeate. The significant parameter towards permeate concentration is based on this following order:

C^2^A > C^2^ > CF^2^ > C^2^F

#### 4.4.2. Average Flux

**Figure 11:**
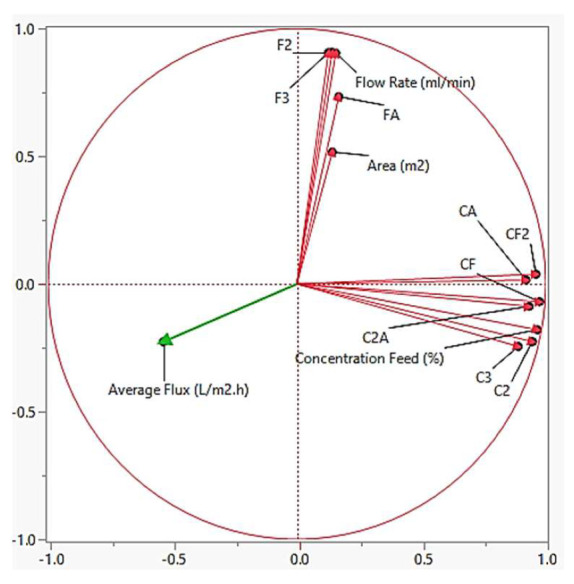
Principle component analysis for the average flux.

The PCA of the average flux is shown in Figure 11. Based on the log worth value in Table 5, it can be concluded that CA has the highest impact towards the model. When plotting PCA, we can see the relationship between the average flux and the crossed variable terms. Based on Figure 11, all of the variables have an inverse relationship with the average flux. For the feed concentration (C), the presence of foulant in the feed controls the fouling effect in membrane separation and thereof affecting the average flux. It can also be seen that variable with only Feed Flowrate (F) (i.e., F^2^ and F^3^) has negative impact towards the average flux. It is with the agreement that lower flowrate creates higher retention time, hence increase the transmembrane pressure which later will increase the average flux. As for the membrane area (A), larger membrane area has lower transmembrane pressure since it takes time for the water to fill in the membrane, hence will give membrane with lower average flux. Moreover, most of the variables have feed concentration (C) in their terms. This can be concluded that the effect of Feed Concentration (C) is more dominant in the cross-variable terms. The significant parameter towards average flux is based on this following order:

CA > C^2^ > C^3^ > C^2^A > CF > FA > CF^2^ > F^3^ > F^2^ > C > F > A

### 4.5. Model Validation

Model validation is carried out to confirm that the generated model is performing closely with respect to the real process. Table 6 and Table 7 shows the model validation for permeate concentration and average flux. Based on Table 6 and Table 7, both conditions have percentage error of ≤5%. This proves that the generated model is 95% accurate and highly feasible. In order to achieve 10 ppm of permeate concentration, the feed concentration, flowrate and area were set at 3.5% of feed concentration with flow rate at 248 mL/min and membrane area of 0.00175 m^2^. The permeate concentration obtained was 10.27 ppm, 3.30% of percentage error with average flux 66.67 L/m^2^ h, 4.45% of percentage error. Meanwhile, another two random numbers were selected, in this case, 69% and 100% of feed concentration with 258.7 and 200 mL/min of flowrate, respectively, with both experiments using same size of membrane area (0.0009 m^2^). The permeate concentration obtained were 1196 and 2517 ppm, which is 2.87% and 5% percentage error from the predicted value of 1162 and 2389 ppm. Meanwhile, the average flux obtained were 50.56 and 197.64 L/m^2^ h with percentage error of 3.22% and 4.98%.

### 4.6. Economic Analysis

#### 4.6.1. Effect of Feed Concentration on Annual CAPEX and OPEX (Rm/y)

**Figure 12:**
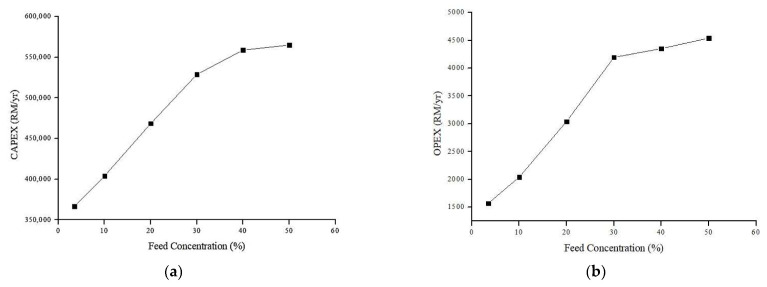
Relationship between annual costs (RM/r) and feed concentration (%): (**a**) Relationship between annual CAPEX (RM/y) and feed concentration (%); (**b**) Relationship between annual and OPEX (RM/y) and feed concentration (%).

The relationship between the feed concentration and the annual CAPEX and OPEX is shown in Figure 12. Other design parameters are held constant, flow rate = 200 mL/min and membrane area = 0.0009 m^2^.

As can be seen from Figure 12, the increase of feed concentration gradually increases both the annual cost. This can be explained by the reduction of permeate flux due to membrane fouling [2,17,65]. The higher number of oil composition/solute in the feed accelerates the pores blockage and cake layer formation hence reduce the amount of permeate [45,66]. Consequently, this increases the specific power consumption (kW/m^3^) which led to higher OPEX as demonstrated in Equation (13). The CAPEX also increases due to increment in the pump price, as can be referred to Equation (7). Since the current permeate flow (m^3^/h) is lower, it causes the scale-up factor to be greater, hence increases the pump flow capacity (m^3^/h), as can be referred to Equation (11) and Equation (7). In other way, higher energy is required to transport water across the membrane.

#### 4.6.2. Effect of Flow Rate on Annual CAPEX and OPEX (RM/y)

**Figure 13:**
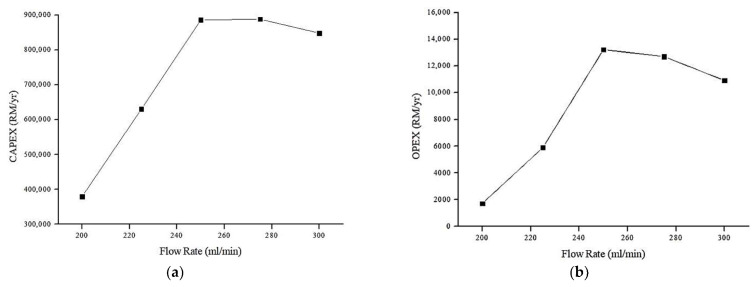
Relationship between annual cost (RM/y) and flow rate (mL/min): (**a**) Relationship between annual CAPEX (RM/y) and flow rate (mL/min). (**b**) Relationship between annual OPEX (RM/y) and flow rate (mL/min).

The relationship between the feed flow rate and the CAPEX and OPEX is shown in Figure 13. Other design parameters are held constant, feed concentration = 100% and membrane area = 0.0009 m^2^.

Based on Figure 13, initially, the flow rate increases both the annual CAPEX and OPEX of the proposed system. This could be related with the relationship of flowrate with specific power consumption of the pump, i.e., higher flowrate, higher power consumption. At lower flowrate, the CAPEX and OPEX is linearly equated. This is due to low average flux in low flowrate region. The fouling effect is more prominent (therefore more energy is required per m^3^ permeate). This is in the agreement with our previous discussion in which the average flux reduces as the flowrate increases due to reduction in membrane contact time with water which led to lower transmembrane pressure. Nevertheless, as the flowrate increases more than 250 mL/min, the average flux increases. It can be assumed that higher feed flowrate would decrease the retention time of the foulant, hence controlling the fouling effect. In other words, higher flowrate reduced the membrane fouling tendency which contributes to higher permeate flux [54]. Therefore, lower energy required per m^3^ permeate.

#### 4.6.3. Effect of Membrane Area on Annual CAPEX and OPEX (RM/y)

**Figure 14:**
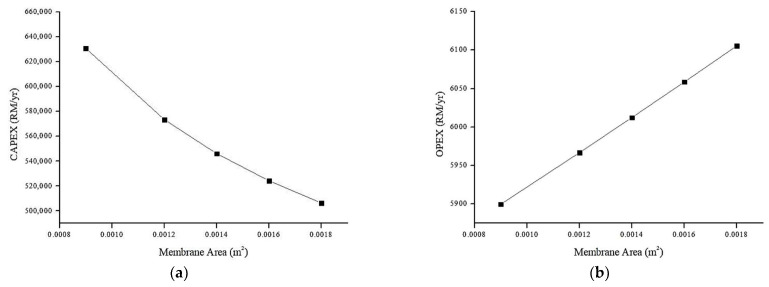
Relationship between annual cost (RM/y) and membrane area (m^2^): (**a**) Relationship between annual CAPEX (RM/y) and membrane area (m^2^). (**b**) Relationship between annual OPEX (RM/y) and membrane area (m^2^).

Figure 14 shows the relationship between the membrane area and the annual CAPEX and OPEX. Other design parameters are held constant, feed concentration = 100% and flow rate = 225 mL/min.

From Figure 14a, the increase in membrane surface area decreases the annual CAPEX. This can be explained by considering the permeate flux and pump price as in Equation (7). From the equation, the pump flow capacity (m^3^/h) is the dominant factor in controlling the pump price considering the pressure is kept constant. Therefore, the higher the membrane area (translated to lower average flux) the lower the pump capacity. Therefore, the CAPEX for the overall system would be reduced. In conjunction, the OPEX increases with membrane area due to lower pump capacity. Larger surface area requires higher specific power consumption (kWh/m^3^) since the permeate flux is lower. This means higher energy is required per m^3^ permeate to achieved targeted permeate water production (100 m^3^/day). Moreover, the membrane replacement price also increases due to the increment in the membrane scale-up factor, as can be seen in Equation (12).

#### 4.6.4. Optimum Design and Scalability

According to Malaysia Environmental Quality Act [67], the minimal discharge quality of oil and grease is 10 ppm. Therefore, the optimum design was based on achieving the minimal discharge quality outline by Malaysia Environmental Quality Act. Based on the scale-up modelling of achieving 10 ppm of permeate concentration with 100 m^3^ permeate volume per day, it is found that the optimum design was 4.9% of feed concentration, 200 mL/min of flowrate and 0.0009 m^2^ of membrane area to achieve 10 ppm oil discharge quality (Table 8). The CAPEX and OPEX of the design are RM 3.742 M and RM/year 1660. 

In scale-up operation, for any membrane technology, membrane fouling is still an issue. It is worth noting that while this study conducted using a small-scale device, fouling was easier to control (by means of high feed flowrate). For scale-up operation, using high flowrate pumping would not be recommended as the OPEX would increase. To solve this issue, it may be more beneficial to dilute the feed concentration via buffer tank prior to treatment based on Figure 2 with respect to effluent discharge quality.

#### 4.6.5. Comparison with Other Literature

Table 9 shows the comparison studies between this proposed system and other membrane filtration systems which focus on oily wastewater treatment. The comparisons are based on the specific energy consumption (kWh/m^3^) and OPEX in terms of utilities (RM/year), extrapolated from literature with respect to calculation in this study. Based on the table, in this study, the proposed MF system has the lowest specific energy consumption (0.09 kWh/m^3^) and lowest OPEX (RM/year 1126) to achieve 10 ppm of oil and grease concentration which is as required by Malaysia Environmental Quality Act [67]. It is then followed by another MF system which requires total OPEX of RM/year 5929 in which flowrate gave major impact on the pump utilities [45]. The highest cost was recorded at OPEX of RM/year 21,346 by Al-Husaini et.al. (2019) which applied UF membrane filtration [68] for synthetic oily wastewater treatment. From this, it can be concluded that the proposed system (cross-flow MF system using waste materials) can be a perfect alternative for PW pre-treatment to reduce the total cost required (reduce the consumption of high energy) while at the same time able to adhere the oil and grease limits as required by the legislation.

## 5. Conclusions

This study explores the potential usage of nylon 6,6 waste polymer which proved that the NFM is feasible to be used for PW treatment. Modelling analysis shows that feed concentration (%) plays a dominant role in controlling permeate concentration (ppm) and average flux (L/m^2^ h) as compared with the flowrate (mL/min) and membrane surface area (m^2^). Moreover, in terms of techno-economic analysis, it can be concluded that permeate flux is the most vital operational factor which has the highest impact in the overall capital and operational costs of this crossflow MF system. The optimum parameters were found at 4.9% of feed concentration, 200 mL/min of flowrate and 9 cm^2^ of membrane area to achieve 10 ppm of permeate concentration with CAPEX at RM 3.7 M and OPEX at RM/y 1660. The significant of this study reports the use of waste NFM in PW treatment using cross-flow MF that is able to achieve standard discharge requirement with attractive economic benefit.

## Figures and Tables

**Table 1:** List of assumptions.

Parameters	Assumptions
Clean Water Production Rate (m^3^/day)	100
Plant operating hours per year (hours/year)	7200
Plant Lifetime (years)	20
Interest rate (%)	8
Pump efficiency	0.8
Electricity Tariff (RM/kWh)	0.441
Power Supply High Voltage (Watt)	30
Time Consumption for Membrane Fabrication (hour)	30
Membrane Area Produced from Spinning (cm^2^)	200
Price of Formic Acid (RM/kg)	21.15
Price of Acetic Acid (RM/kg)	0.000423

**Table 2:** Factors included for capital cost calculations [51].

Parameters	Assumptions	Factor
f_1_	100	0.4
f_2_	7200	0.7
f_3_	20	0.2
f_4_	8	0.1
	Physical Plant Factor	1.4
f_5_	0.441	0.3
f_6_	30	0.05
f_7_	Contingency Cost	0.1
	Fixed Capital Factor	0.45

**Table 3:** Membrane properties of nylon 6,6 waste NFM.

Sample Name	Nylon 6,6 Waste
Thickness (mm)	0.29 ± 0.05
Porosity (%)	81.34
Pore Size (µm)	0.20
Fibre Diameter (nm)	104.65 ± 65.49

**Table 4:** The logworth and *p*-value data for concentration permeate.

Source	Logworth	*p*-Value
Feed Concentration (%) × Feed Concentration (%) × Area (m^2^) (C^2^A)	8.540	0.00000
Feed Concentration (%) × Feed Concentration (%) (C^2^)	7.603	0.00000
Feed Concentration (%) × Flow Rate (mL/min) × Flow Rate (mL/min) (CF^2^)	6.847	0.00000
Feed Concentration (%) × Feed Concentration (%) × Flow Rate (mL/min) (C^2^F)	4.668	0.00000

**Table 5:** The logworth and p-value data for average flux.

Source	Logworth	*p*-Value
Feed Concentration (%) × Area (m^2^) (CA)	6.850	0.00000
Feed Concentration (%) × Feed Concentration (C^2^)	4.979	0.00001
Feed Concentration (%) × Feed Concentration (%) × Area (m^2^) (C^2^A)	3.885	0.00013
Feed Concentration (%) × Feed Concentration (%) × Feed Concentration (%) (C^3^)	1.972	0.01067
Feed Concentration (%) × Flow Rate (mL/min) (CF)	1.498	0.03179
Flow Rate (mL/min) × Area (m^2^) (FA)	1.466	0.03423
Feed Concentration (%) × Flow Rate (mL/min) × Flow Rate (mL/min) (CF^2^)	1.405	0.03938
Flow Rate (mL/min) × Flow Rate (mL/min) × Flow Rate (mL/min) (F^3^)	0.866	0.13603
Flow Rate (mL/min) × Flow Rate (mL/min) (F^2^)	0.766	0.17120
Feed Concentration (%) (C)	0.669	0.21440
Flow Rate (mL/min) (F)	0.640	0.22928
Area (m^2^) (A)	0.044	0.90366

**Table 6:** Model validation for concentration permeate.

Source	Feed (%)	Flowrate (mL/min)	Area (m^2^)	Predicted	Experimental	Percentage Error (%)
1	3.5	248	0.00175	9.94	10.27	3.30
2	69	258.7	0.0009	1196.14	1161.79	2.87
3	100	200	0.0009	2517.17	2389.26	5.00

**Table 7:** Model validation for average flux.

Source	Feed (%)	Flowrate (mL/min)	Area (m^2^)	Predicted	Experimental	Percentage Error (%)
1	3.5	248	0.00175	69.77	66.67	4.45
2	69	258.7	0.0009	48.98	50.56	3.22
3	100	200	0.0009	208	197.64	4.98

**Table 8:** Optimum parameters for scale-up MF.

Conc. Feed (%)	Flow Rate (mL/min)	Area (m^2^)	Average Flux (L/m^2^h)	Power Consumption (kWh/m^3^)	Con Permeate (ppm)	CAPEX (RM)	OPEX (RM/y)
4.9	200	0.0009	216.5	0.09	10	3.743 M	1660

**Table 9:** Comparison studies on membrane filtration for oily wastewater treatment in terms of OPEX (RM/year).

No	Type of Membrane	Flowrate (mL/min)	Membrane Area (m^2^)	Feed Conc (ppm)	Permeate Conc (ppm)	Average Flux (L/m^2^ h)	Specific Energy Consumption (kW/m^3^)	OPEX in Terms of Utilities (RM/y)	Ref.
1	MF	200	0.0009	304.13	10	217	0.09	1126	This study
2	MF	237.6	0.0009	88.43	4.93	80	0.45	5929	[45]
3	UF	1000	0.0025	12,000	660	347	1.61	21,346	[68]

## Data Availability

Data are unavailable due to privacy.

## Nomenclature

ANOVA	Analysis of Variance
CAPEX	Capital Cost/Capital Expenditure
FESEM	Field Emission Scanning Electron Microscope
FO	Forward Osmosis
FTIR	Fourier Transform Infrared Spectroscopy
MD	Membrane distillation
MF	Microfiltration
MVC	Mechanical Vapor Compression
NF	Nanofiltration
NFM	Nanofiber Membrane
OPEX	Operating Cost/Operating Expenditure
PCA	Principle Component Analysis
PPC	Physical Plant Cost
PW	Produced Water
RMSE	Root Mean Square Error
RO	Reverse Osmosis
TEA	Technoeconomic Analysis
UV-Vis	Ultraviolet-Visible
WCA	Water Contact Angle

## Appendix A

**Table A1:** Design of experiment for cross-flow microfiltration (MF).

Run	Feed Concentration (%)	Flow Rate (mL/min)	Area (m^2^)	Permeate Concentration (ppm)	Rejection (%)	Average Flux (L/m^2^ h)
1	0	200.00	0.0009	0.00	0.00	214.44
2	25	225.00	0.0009	100.20	79.78	79.78
3	25	275.00	0.0009	67.98	86.28	101.11
4	50	200.00	0.0009	928.27	57.75	78.00
5	50	250.00	0.0009	1008.48	54.09	87.22
6	50	300.00	0.0009	1122.17	48.92	88.33
7	75	225.00	0.0009	1461.21	60.47	61.67
8	75	275.00	0.0009	967.22	73.83	64.00
9	100	250.00	0.0009	2097.84	66.20	44.67
10	0	225.00	0.0018	0.00	0.00	82.22
11	25	225.00	0.0018	217.70	56.08	25.28
12	25	275.00	0.0018	286.94	42.10	21.11
13	50	250.00	0.0018	508.13	76.87	59.44
14	50	300.00	0.0018	558.27	74.59	17.22
15	75	225.00	0.0018	861.49	76.69	79.07
16	75	275.00	0.0018	928.27	74.89	24.50
17	100	250.00	0.0018	876.53	85.88	65.00
